# Patient Derived Ex-Vivo Cancer Models in Drug Development, Personalized Medicine, and Radiotherapy

**DOI:** 10.3390/cancers14123006

**Published:** 2022-06-18

**Authors:** Ryan Zitter, Rishi Man Chugh, Subhrajit Saha

**Affiliations:** 1 Department of Radiation Oncology, University of Kansas Medical Center, Kansas City, KS 66160, USA; rzitter@kumc.edu (R.Z.); rchugh@kumc.edu (R.M.C.); 2 Department of Cancer Biology, University of Kansas Medical Center, Kansas City, KS 66160, USA

**Keywords:** patient-derived organoids, organ-in-chip, cancer models, drug development, personalized medicine, radiotherapy

## Abstract

**Simple Summary:**

This review article highlights gaps in the current system of drug development and personalized medicine for cancer therapy. The ex vivo model system using tissue biopsy from patients will advance the development of the predictive disease specific biomarker, drug screening and assessment of treatment response on a personalized basis. Although this ex vivo system demonstrated promises, there are challenges and limitations which need to be mitigated for further advancement and better applications.

**Abstract:**

The field of cancer research is famous for its incremental steps in improving therapy. The consistent but slow rate of improvement is greatly due to its meticulous use of consistent cancer biology models. However, as we enter an era of increasingly personalized cancer care, including chemo and radiotherapy, our cancer models must be equally able to be applied to all individuals. Patient-derived organoid (PDO) and organ-in-chip (OIC) models based on the micro-physiological bioengineered platform have already been considered key components for preclinical and translational studies. Accounting for patient variability is one of the greatest challenges in the crossover from preclinical development to clinical trials and patient derived organoids may offer a steppingstone between the two. In this review, we highlight how incorporating PDO’s and OIC’s into the development of cancer therapy promises to increase the efficiency of our therapeutics.

## 1. Introduction

From the time of its inception in a research laboratory, delivering a cancer therapeutic to a patient often takes about fifteen years [1]. Even after this rigorous process of laboratory and clinical research, patient specific differences in treatment response limits therapeutic benefit significantly [2]. This disconnect between preclinical research and the bedside can be attributed to the models we have used to develop these therapeutics.

Specifically, there are two separate situations that require more physiologically accurate models. The first is drug screening and validation as part of a developmental process that requires translationally relevant models to reduce false positive drug candidates. Next, determination of personalized differences in the treatment response is also important to predict therapeutics’ efficacy. It is critical that models used in these two processes are able to physiologically replicate the tissue they are derived from and can also be replicable between academic institutions. An effective model is one that is scalable, personalized, and able to mimic human physiology.

Immortalized human cell lines with a two-dimensional culture system have proven useful in therapeutic development but inherently fall short of having bedside prediction. In the case of immortalized human cell lines, known patterns of genetic expression are useful in target optimization but require testing across a multitude of cell lines to determine any unforeseen molecular interactions. Conditionally reprogrammed patient-derived samples have been successfully converted into a two-dimensional culture, thus adding genetic diversity to the lines of cells tested [3]. However, this does not avoid cells being cultured in two-dimensional settings under poorly modeling the physiologic environment. Standard monocellular culture generally fails to capture the complex states of hypoxia within tumors [4], stromal impact [5], or immune system interactions [6], which are known to have a therapeutic impact in the clinic. 

In this facet, patient-derived xenograft (PDX) models are a step closer in recreating physiological conditions. However, PDX’s predictive weakness stems from genetic change driven by mouse implantation [7,8]. This mismatch of gene expression can ultimately misguide therapeutic data tested in these models. Furthermore, PDX models often require immunocompromised hosts, inhibiting the investigation of immune system interaction—though humanized mouse models may offer a work around [9].

It is also worth mentioning the progression of in silico models to this point. Processing high volume cancer genome datasets has enabled the identification of new driving mutations for therapeutic development [10]. In the realm of personalized medicine, mathematical models can be used to predict tumor response and therefore adapt the timing of therapeutic dosing to maximize efficacy [11]. Mathematical modeling is also specifically applicable to radiotherapy [12]. While cost efficient and complimentary to in vitro models, in silico models are inherently unable to capture a real-world response to therapy.

**Figure 1:**
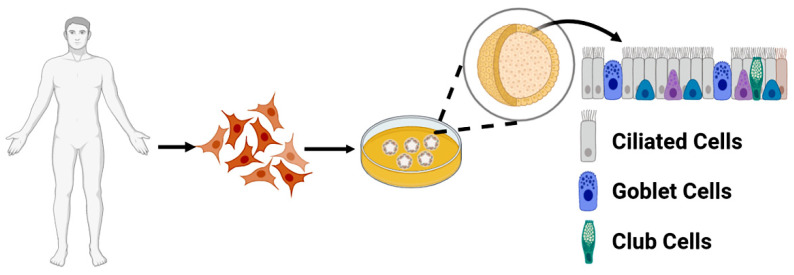
Diagram demonstrating the morphology of an organoid model. Tissue biopsies containing stem cells (in this case patient-derived ASCs) are able to be extracted and then cultured in a Matrigel that allows the organoid to take its three-dimensional form. The organoid will differentiate into the different cell types of its native tissue, in this case intestinal epithelium. Intestinal epithelium consists of multiple cell type tissue containing intestinal stem cells, transit amplifying cells and differentiated cells (ciliated cells, goblet cells, and club cells).

Organoid-based models have begun to address some of these shortcomings. The fundamental basis of an organoid is that of a cluster of cells derived from a stem cell that self-renews and differentiates into several functional cell types with a morphology representing their tissue/organ-of-origin (Figure 1). The possibility of regenerating these “mini organs” from single cells was recognized as early as 1907 [13] and could be reproduced animal embryonic stem cells (ESCs) in the 1940s [14]. The same effect can be achieved using induced pluripotent stem cells (iPSCs) or adult stem cells (ASCs).

It was in the 1980s that groups began to understand the effect of media conditions on organoid culture. Li et al. demonstrated that breast epithelial cells were able to form ducts and produce milk given the right extracellular matrix, where 2D media failed to form any milk [15]. The watershed moment in organoid models came in 2009 when Sato et al. first established long-term organoid cultures from a single ASC [16]. After murine intestinal Lgr5+ cells were isolated, these highly proliferative cells established crypt-villus organoids resembling the structure of their native tissue.

**Figure 2:**
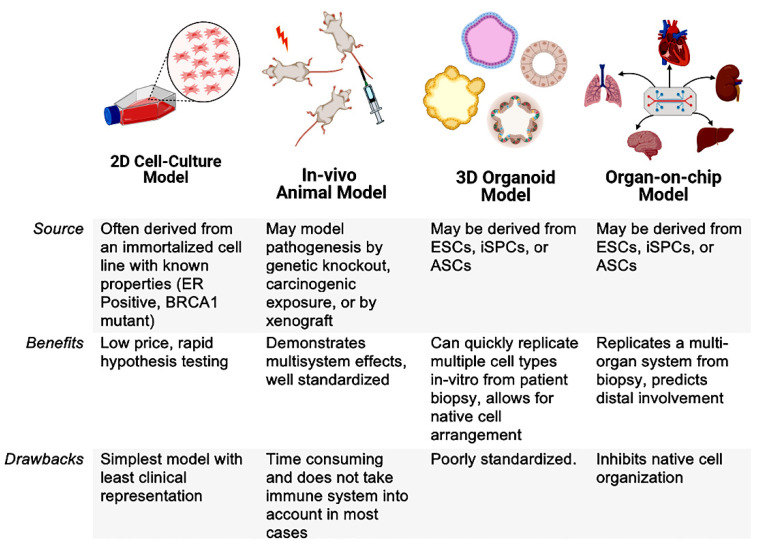
The range of models currently available in oncology with inherent drawbacks or advantages for desired research questions.

This replication of native tissue morphology and cellular differentiation is fundamental in observing a response to treatment. The range of tissue specific cell types and three-dimensional structure achieved in organoids better represents the cellular interplay within the human body. Highly complicated processes such as angiogenesis [17] and neuronal organization [18] are able to be replicated due to the self-organizing process of these stem cells. This self-organization results in cell proliferation and gene expression levels that more closely mimic a cell’s native organ than it would when in 2D cell culture [19]. Therapeutic evasion is also demonstrated to be better modeled in organoid platforms, as stromal signaling impacts tumor resistance [20]. Even the hypoxic tumor environment is able to be modeled, as organoids can mimic hypoxia gradients [21]. Recent advancements have elevated the complexity of organoid models even farther, to be multi-tissue based. These systems—called organ-in-chip (OIC)—extends the spectra of available models (Figure 2). As each model type has its select advantages, it is important to consider the benefits and drawbacks of each system. Being the most advanced does not necessarily make it the most appropriate for any given research question being asked.

## 2. Patient-Derived Organoids

Patient-derived organoids (PDOs) are, most simply, an organoid culture established from stem cells derived from patient biopsy. This can include tumor or normal tissue samples. Since the first established murine culture in 2009, a multitude of disease pathologies have been successfully established in human tissues using this model [22]. To date, normal tissue PDO cultures have been established in colorectal [23,24], hepatocellular [25], gastric [26], prostatic [27,28], trachea [29], kidney tubule [30], and fallopian tube tissues [31]. Malignant tissues with established PDO cultures include biliary [32], pancreatic [33], and breast [34,35]. Furthermore, PDOs mimicking metastasis have been established in settings such as colorectal peritoneal masses [36].

Patient-derived organoids have been demonstrated to be superior to two-dimensional culture and PDX in several consequential areas. The first is the maintained degree of genetic similarity to the original biopsy [37]. Leveraging this genetic stability, great genomic diversity can be achieved using a library of PDOs (discussed later) and results in a more varied pharmacological response. This can be leveraged to reveal new genomic links to drug resistance [38]. Furthermore, enzymes such as CYP3A4 are expressed near levels found in vivo, a crucial aspect in drug development when CYP3A4 is estimated to account for 45% of phase one drug metabolism in humans [39,40]. When inspecting drug metabolism grossly, organoids have been shown to correlate with the half maximal inhibitory concentration observed in clinic [41]. Maintaining these enzymatic expression levels and drug metabolism in a more true-to-human model avoids the model deviation found in PDXs. These features have led to organoids being useful across specialties including cardiology [42,43], toxicology [44], bacterial pathogenesis, and most recently COVID-19 research [45,46].

## 3. Organoid Limitations

While organoids better model the morphology of their native tissue, the absence of stroma and microenvironment significantly limits their translational relevance. Inclusion of stromal niche to mimic the micro-environment is very critical to study the complex tissue system, such as the tumor. Tumors have a complex microenvironment, including dense extracellular matrix, stromal, immune, and irregular vascular infiltrate [47,48]. It has been indicated that colorectal tumor growth is maintained by immune-related factors and other stromal components [49]. Therefore, creating a model without stromal components may completely fail to capture the driving components of the tumor.

The ex vivo modeling of immunotherapies is difficult for this exact reason and possibly why immunotherapy has had such mixed clinical results [50]. Adapting the specificity of the PDO model to the complexity of the immune response is certainly difficult, but promising attempts have been made. Non-small cell lung (NSCL) and colorectal cancer (CRC) organoids have both been studied by culturing these tissues with peripheral blood lymphocytes [51]. Tissue native intraepithelial lymphocytes (IELs) have also been cocultured in both intestinal and pancreatic tissue and demonstrate cytokine signaling and lymphocyte motility [52,53]. Studying IELs is particularly exciting, as they may offer a preventative immunosurveillance approach [54]. Though not yet capable of maintaining a true PDO due to the complex nature of the extracellular matrix, encouraging steps have been taken towards creating thymus organoids [55,56]. There is certainly a path forward in enriching PDO models with an immune system coculture, but this still fails to capture the patchwork of intracellular relationships within the body.

For instance, irritable bowel syndrome (IBS) is a disease that extends beyond the complexity of intestinal epithelial PDO models. The intestinal response in IBS mimics one of infection or inflammation and leads to a cascade of inflammatory cells, edema, and release of cytokines [57]. Furthermore, the interplay of a microbiome–gut–brain axis is known to play a role in the disease [58,59]. This constellation of processes illustrates just how hard it may be to model intestinal disease and how our cancer models may very well be missing tissue-defining mechanisms. For these multi-organ-system etiologies, a more complex model such as organ-in-chip is required to model a patient with accuracy.

The extracellular matrix is another important variable in organoid culture. The hydrogel scaffold used to support organoid growth can cover a wide range of stiffnesses that introduce a variety in cell differentiation [60]. Commercial hydrogels are for the most part animal-derived and not modifiable for tissue specific needs, though work is being conducted in the development of synthetic gels [61]. Emulating the extracellular matrix of the native organ may prove to be even more complicated when considering tissue co-culture.

## 4. Organ-in-Chip

**Figure 3:**
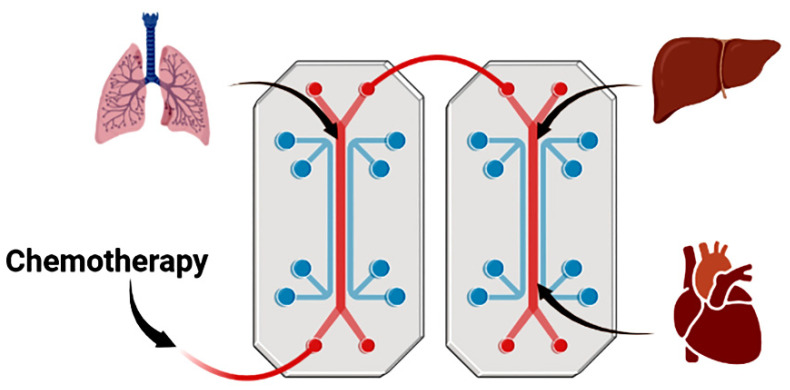
Schematic representation of organ-in-chip system, in which tissue biopsy from multiple organs is connected by microfluidic channels allowing molecular communication. This creates a model of distal interactions and multi-organ axis. In this instance—a chemotherapy agent can be tested for its effect on lung tissue, while monitoring how secondary messengers interact with liver and lung tissue. Organ-in-chip technology allows detection of the metabolic profile involving multi-organ syndrome due to side effects.

In more complex disease systems such as those mentioned, utilizing organ-in-chip (OIC) acts as a system to utilize the necessary components of a good model: scale, personalization, and mimicry [62]. OIC models incorporate fluid motion and multiple tissue types to grow multiple organoids in tandem (Figure 3) [63]. Just as the surrounding stroma plays a role in cell formation and signaling, cellular movement influences cellular growth and pathology—such as peristalsis in intestinal epithelium [64]. Even the chip surfaces may be altered to best mimic heterotrophic tissues in vitro, such as the liver [65]. Beside basic etiology research in multi-organ pathologies such as IBD, these dynamic systems are useful in testing first pass pharmacokinetics such as MDR1 efflux effects in the intestine [66,67]. The assessment of lumen integrity and drug diffusion in OIC has also be used to study drug efficacy [68,69].

OIC is also unique in offering a means of studying distal organ interactions. A unique example of this is the hepatocellular/testis model developed by Baert et al. that was used to study male repro-toxicity [70]. Studying organoid drug response in series enables researchers to study the effect of hepatocellular metabolized drug on the testes as well as the hepatocellular response to changes in testis hormone production. A similar model of distal organoids connected via OIC in series has also been designed utilizing a liver/heart/lung system [71].

In addition to these unique insights on drug development and toxicity, the organ-in-chip offers a means of studying metastatic tumorigenesis and prevention. Hemodynamic force facilitates the transport of bloodborne metastasis and is therefore a pathology well suited to be studied in OIC models. Mimicking blood flow on the chip, it is possible to label metastatic cells to determine the extent to which they encounter rolling adhesions in the same way they would distally attach in vivo [63,72]. Distal organ metastasis models have been successfully created to study pharmacologic impact on migration, opening the door for the same to be achieved in patient-derived samples in personal medicine [73]. In a technically complex but conceptually straightforward experiment, Aleman and Skardal studied colorectal metastasis preference using a four-organoid model [74]. Systems such as these open possibilities to pre-screen and validate toxicity and treatment efficacy in a patient-specific personalized basis.

One drawback to OIC modeling is pointed out by Miura et al., in that it inherently limits cellular self-organization [20]. In their “assembloid” model, human pluripotent stem cells are converted into brain region specific organoids and are allowed to integrate and form complex cell-to-cell interactions. This can also be observed in the previously mentioned Palikuqi et al., where allowing endothelial cells to grow with organoids allows for the formation of functional vasculature [17].

## 5. Utilization of Ex Vivo Models in Cancer Therapy

**Figure 4:**
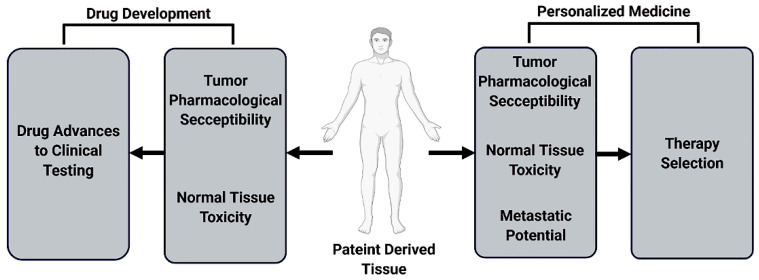
Patient-derived organoids have a significant role in drug screening, validation, development, and in personalized medicine. On a high throughput scale, biobank organoids can capture a wide swath of diversity for pharmaceutical development. On the individual level, paired organoid studies could help guide therapeutic selection.

These progressive models—PDO and OIC—are well poised to play two separate but related roles in cancer therapy. The first is drug development. Using large scale biobanks of PDOs, high throughput testing can break away from the shortfalls and inefficiencies of 2D culture and PDX, as discussed previously. The second and most exciting application is real-time personalized medicine (Figure 4).

## 6. Drug Development

Becoming more efficient in our preclinical models in oncology is imperative, as the field faces soaring drug development costs [75] and an abysmal phase I approval rate estimated to be about 5% [76]. Improving the preclinical stage with patient-derived modeling could reduce the number of inadequate drugs that are advanced to the clinical phase and free up industry resources. This is possible using biobanks of PDOs conserved under long term culture [77], though some tissues still pose challenges in achieving this maintainability [78]. One such biobank has been established in gastric cancer, accounting for wide molecular diversity across normal, dysplastic, cancerous, and metastatic tissue [79]. Similar long term and genetically heterogenous PDO libraries have been described in colorectal [80], breast [35], and bladder tissue [81].

Verissimo et al. utilized a biobank such as this to select a subset of RAS mutant colorectal cancers to test combinational drug testing demonstrating the potential of this system [82]. Testing combination therapies is particularly important in cases of acquired resistance to target therapies such as RAS. High throughput systems enable testing thousands of possible combinations while reducing time.

**Figure 5:**
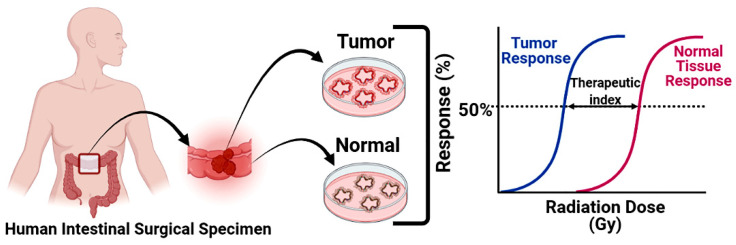
Paired organoid system (POS) to determine radiation response to both normal and malignant tissue collected from same organ. POS can determine therapeutic index of radiation in ex vivo in a personalized basis.

A crucial component of drug development that could be streamlined with these models is normal tissue toxicity investigation. Early phase clinical trials could greatly benefit from studying drug effects in patient-derived systems—and could very well prove more enlightening than the industry standard animal testing. Utilizing already existing organoid biobanks to evaluate toxicity in this way could prevent inappropriate drug advancement while minimizing animal costs. We have demonstrated that the patient-specific same-organ paired organoid system (Figure 5) can be used to examine the normal and malignant tissue specific response of radio-modulators [83,84]. This paired system allows for the expedited assessment of not only normal tissue toxicity in the case of antineoplastic agents [85], but also any preneoplastic side effects of protectant agents.

An equally important preclinical metric of pharmaceutical development is that of absorption and excretion. One existing OIC duodenum model gives insight into drug effect on permeability, microvilli, and tissue morphology [66]. Similar studies have been conducted on hepatic tissue, a key in drug metabolism and toxicity [86]. Herland et al. d in a marrow–liver–kidney OIC platform that they were able to recreate organ specific toxicity dosing of cisplatin found in vivo [87]. This suggests that OIC could find pharmacodynamic concerns before accumulating the costs of phase I trials.

Furthermore, the OIC model, in particular among ex vivo models, offers an advantage in understanding the tumor microenvironment’s influence during drug development. The degrees of freedom offered in this model—timing of pulsatile signaling, rate of fluidic streams, or timing of toxin exposure—allows for the identification of biokinetic influences. For instance, altering electrical stimulation of cardiomyocytes in vitro greatly dictates cell maturation and therefore response to pharmaceuticals [88]. Multiarmed OIC trials in variations of the microenvironment paired with high throughput OIC assessment, as showcased by Peel et al., offers a highly efficient means of pinpointing drug candidates [89].

As biobanks begin to grow, it will be important to create multiple biopsy profiles to take advantage of multisystem models such as OIC or paired organoids. Creating a high throughput system of biopsy and subsequent OIC culturing would be the ultimate preclinical trial in therapeutics and could revolutionize industry efficiency.

Lastly, a dimension of drug development that can benefit from PDO models is the novel biomarker discovery. Culture media of known organoid pathologies can be matched with patient plasma to aid in diagnosis [90] and in determining drug response. As the role of miRNA signaling becomes a clearer target for therapeutics, organoids will be invaluable as they are able to replicate these signaling cascades in vitro [91].

## 7. Personalized Medicine

While organoids demonstrate obvious advantages in drug development and basic research, their application has the most revolutionizing impact on personalized medicine. Currently, there are 586 FDA approved anti-cancer drugs [92]. With such a library of already existing therapies, pairing a patient to a treatment is the primary goal of personalized medicine. Who could better predict a patient’s response better than the patient’s own tissue? Patient-derived models that are streamlined to return therapeutic data before beginning treatment can and have been highly effective in maximizing the patient response.

Genotypic onco-typing has already acted as an oncologist’s greatest guidance in selecting therapy—especially as more therapies have come to market. Clinical trials have trudged forward to prove which doses, drug combinations, and therapies are most effective through statistical power and large numbers. Massive trials such as the MAGRIT trial, which has enrolled 2661 patients across some 400 centers in 33 countries, have pushed the envelope in capturing massive amounts of the effected population [93,94]. These datasets have great value in proving the efficacy of a drug in a diverse population but does little to tell a physician whether a treatment is best for the patient sitting in front of them. The idyllic personalized model would use patient biopsies to guide treatment choice within the guardrails of these large studies.

Indeed, there are already cases in which PDO and OIC systems have been able to guide treatment. Loong et al. reported the case of a 58-year-old male diagnosed with glioblastoma multiforme (GBM), where patient-derived organoids were used to guide treatment [95]. Using genome-guided candidates, a drug panel testing was performed on these organoids. Notably, temozolomide resistance in this tissue was predicted in the PDO model. After mTOR inhibitor everolimus and MEK inhibitor cobimetinib demonstrated enhanced cytotoxicity, everolimus was chosen as the therapeutic course due to concerns of cobimentinib efflux in GBM [96]. After four weeks of treatment, the imaging showed reduced mass effect. After a forced break in treatment, resumption of everolimus demonstrated interval reduction in size and contrast enhancement, with significant reduction in edema and mass effect. Loong et al. cited this response to illustrate the tumors’ dependence on the PTEN pathway. A similar vignettes of parallel organoid trials in cancer is described in papillary carcinoma [97].

The importance of the parallel PDO model as a preliminary study of therapeutic response is highlighted in ovarian cancer organoids [98]. In this study, stalled replication fork defects were present in 61% of tested organoids, while only 6% had functional homologous recombination and PARPi sensitivity. Isolating mechanisms of chemosensitivity rapidly after diagnosis is key in efficient treatment.

Cystic fibrosis (CF) management has also demonstrated the impact PDOs can have on choosing a treatment. In a cohort of 24 CF patients, patient-derived rectal organoids were used to pair in vitro and in vivo responses [99]. The PDO’s cultured correlated with both a change in pulmonary response and change in sweat chloride concentration. These changes correlated with a clinical change in both the pulmonary and sweat chloride response. The authors of the study further suggested that thresholds could be established in their assay to easily identify responders in a cost-effective way.

Again, in oncology, Narasimhan et al. sequenced and drug panel-tested PDOs in 28 patients with colorectal cancer with peritoneal masses (CRPMs) [36]. PDOs were successfully established and profiled in 19 out of 28 patients within 8 weeks. Drug panel results were presented to the medical oncology team upon the failure of standard care. This resulted in treatment change for two patients, one of whom had a partial response despite previously progressing on multiple rounds of standard chemotherapeutics. This predictability of PDO has been verified in another colorectal trial [100] as well as in prostate [101], serous ovarian [102], pancreatic [103], and papillary thyroid [104] cancers.

On a larger scale, A phase II trial of the Aurora Kinase A inhibitor alisertib was conducted [105]. Patient-derived organoids grown in parallel with patient treatment exhibited concordant responses to alisertib, demonstrating the predictive value of PDOs in prostate cancer. Ongoing clinical trials utilizing a parallel PDO arm to predict response include the RAMONA trial [106] and the STRONG trial [107].

In order to improve the rate of development in new therapeutics, the National Center for Advancing Translational Sciences (NCATS), in conjunction with several other National Institutes of Health (NIH), has recently funded ten projects utilizing OIC to inform clinical trial planning [108]. Current projects range from non-alcoholic fatty liver disease to treatment of catecholaminergic polymorphic ventricular tachycardia. In oncology specific trials, one of these projects’ models castrate-resistant prostate cancer utilizing OIC to recreate the bone marrow microenvironment [109].

As oncology progresses towards the goal of personalized medicine, validated ex vivo models stand to be a unique tool in clinicians’ hands. With the integration of the biopsy into the cancer staging process, PDO or OIC response can only add information for treatment guidance.

## 8. Ex Vivo Models in Radiotherapy

Of the oncologic subspecialties—radiation oncology is uniquely situated to benefit from patient-derived modeling. Progress in the field of radiation oncology has chiefly focused on anatomical precision to reduce these acute side effects. MRI-guided radiotherapy in gynecologic cancers can reduce exposure to adjacent organs by up to 59% [110]. Proton beam therapy also reveals marked reduction in an off-target dose due to its inherently confined Bragg peak distribution [111,112]. While this has reduced side effects of treatment, empiric dosing and fractionation are still cornerstones of the field. Tumor response to radiotherapy can be highly heterogenous. Neoadjuvant chemoradiotherapy in esophageal carcinoma may result in a complete response (25% of patients), partial response (55%), or no response (20%) [113,114]. The use of pre-treatment imaging in radiomics [115,116] and patient-derived plasma samples is beginning to be used in the search for radiation biomarkers [117] to predict treatment response. However, patient-derived organoid models in development of predictive biomarkers or screening of radio-modulators can be a very useful tool. The predictive value of organoid models in patient-derived tumors has been validated in rectal cancers [118]. Incorporating this type of information in the pretreatment workup can greatly improve clinicians’ choice in tumor dosing.

Equally important to predicting an individual’s tumor response and their normal tissue radiotoxicity. Unplanned breaks in radiotherapy treatment due to toxicity can have significant negative effect on treatment outcomes [119,120]. Differences in inherent radiosensitivity—a concept long known about but hard to clinically account for—is a pitfall that organoids are perfectly suited to predict. Even within the realm of precision radiotherapy, understanding the individual patients response to both tumor and normal tissue stands to make treatment more effective. A multi-organ paired PDO model would be uniquely suited in determining the response of both tissue types even with heavy ion and proton beam therapies. In certain tumor types, biopsy of anatomically neighboring organs for multi-organ ex vivo modeling would identify both normal tissue toxicity and tumor response. For instance, pancreatic tumors cannot be discussed as candidates for radiotherapy without mention of duodenal toxicity [121]. Identifying patients who may or may not benefit from these therapies is especially important now as access to these therapies is limited.

## 9. Conclusions

This constellation of data draws a picture of two sectors of medicine that stand to gain from translational science: Drug development and personalized medicine. The data offered by a patient’s own tissue is invaluable both in the lab and clinic and should be leveraged as such. Working smarter—not harder—to make preclinical drug development more efficient and making therapeutic choices more accurate stands to make oncology more affordable and more effective.
